# Correlation between serum bile acids with severity of liver cirrhosis: A case-control study

**DOI:** 10.6026/973206300213005

**Published:** 2025-09-30

**Authors:** Soham Doshi, Pratibha Sonawane, Uditkumar Agrawal, Ashfiyah Altafhusen Munshi

**Affiliations:** 1 Department of General Medicine, Government Medical College and Maharashtra Post Graduate Institute of Education And Research MUHS, Nashik, Maharashtra, India; 2 Department of Biochemistry, NSC Government Medical College Khandwa, Madhya Pradesh, India; 3 Department of Pathology, GMERS Medical College, Himmatnagar, Gujarat, India

**Keywords:** Serum bile acids, liver cirrhosis, child-pugh score, MELD score, biomarkers, liver function tests, disease progression

## Abstract

The association of serum bile acid levels with liver cirrhosis severity is of interest. Hence, 100 patients were admitted, among them 50 patients had variable severity of liver cirrhosis and 50 patients were controls. Serum bile acid levels were measured and their association was also analyzed with clinical parameters like Child-Pugh score, MELD score and liver function tests. The results indicate that serum bile acid concentrations are strongly associated with the severity of liver cirrhosis and potentially as a disease progression biomarker. Thus, the clinical value of bile acids as a marker for the severity of liver cirrhosis is shown.

## Background:

Liver cirrhosis is an irreversible and progressive liver illness with extensive fibrosis and hepatic failure. It represents the ultimate stage of chronic liver diseases, and it has multiple causes including viral infections (hepatitis B, C), alcoholism, non-alcoholic fatty liver disease (NAFLD), and inherited diseases like hemochromatosis [1]. Cirrhosis leads to potentially lethal complications such as portal hypertension, hepatic encephalopathy, and liver failure. Early detection and proper staging of cirrhosis are of absolute value in the treatment of the disease, follow-up of disease progression and planning of appropriate therapeutic maneuvers [2]. Cirrhosis stage is typically established using clinical criteria, especially the Child-Pugh score and the MELD score, which are determined by liver function tests and clinical aspects such as ascites, encephalopathy and bilirubin levels [3]. However, these scoring systems are plagued by failing to reflect accurately the underlying pathophysiological changes in liver function. Hence, there is growing interest in establishing confident biomarkers that not only can be employed as predictors of disease severity but also provide insight into the pathophysiology of cirrhosis [4]. One such biomarker is serum bile acids, which are crucial in the digestion and absorption of dietary lipids and fat-soluble vitamins. Bile acids are synthesized in the liver from cholesterol and normally excreted into the bile and stored in the gallbladder [5]. Liver impairment in cirrhosis leads to an imbalance of bile acid metabolism, leading away from normal serum bile acid levels. Bile acid deposition in the blood has been linked to cholestasis and hepatocyte injury and recent studies have shown that alterations in bile acid profiles may be used as an index of the severity of liver injury and fibrosis [6]. Earlier work has emphasized the involvement of bile acids in liver disease by demonstrating that some bile acid species can be used as markers for liver dysfunction. Elevation of serum bile acids in cirrhotic patients has been correlated with the severity of the liver disease and hence these molecules not only indicate hepatic damage but could be participating in the fibrotic process as well [7]. The precise mechanisms for the changed bile acid levels in cirrhosis are still poorly defined, and their clinical value as markers of the severity of cirrhosis still needs to be fully established [8]. Therefore, it is of interest to explore the correlation between bile acid levels in the serum and the degree of liver cirrhosis in a group of patients with various phases of the disease.

## Materials and Methods:

This research was carried out at [Hospital/Institution Name], [Location], between [start month, year] and [end month, year], on 100 patients. The patients were assigned to two groups: 50 liver cirrhosis patients and 50 healthy controls. Liver cirrhosis in the study group was diagnosed on the basis of clinical manifestations, biochemical examination, imaging modalities (ultrasound, CT, MRI) and, whenever required, liver biopsy or elastography. The control group was made up of healthy subjects without liver disease, chronic alcoholism, or any systemic illness. Patients enrolled in the study presented with cirrhosis due to prevalent etiologies such as chronic viral hepatitis, alcohol use, or NAFLD. Patients were not recruited if they had secondary cirrhosis due to autoimmune hepatitis, liver cancers, or other non-cirrhotic liver diseases. Exclusion was also based on being pregnant, below 18 years, acute infection, or any comorbidities affecting bile acid metabolism or liver function, including renal or cardiovascular disease. Demographic and clinical data were collected in both groups, such as age, sex, history of disease and signs of liver disease such as ascites, jaundice, and encephalopathy. Blood was taken after 12 hours of fasting and biochemical parameters were assessed, such as liver and kidney function tests (ALT, AST, bilirubin, albumin, INR, GGT, ALP). Serum bile acids were quantitated by high-performance liquid chromatography (HPLC) or liquid chromatography-mass spectrometry (LC-MS/MS), whichever was available in the laboratory. The Child-Pugh score and MELD score were used to quantify the severity of liver cirrhosis among the participants in the study. The Child-Pugh score using the level of bilirubin, albumin and presence of ascites, encephalopathy, and prothrombin time was utilized to grade cirrhosis as class A, B, or C. The MELD score, being a scoring system that is used to measure the severity of cirrhosis and for the prediction of short-term mortality, was also calculated. Both these scoring systems were utilized to match with the serum bile acid levels. For inferential statistics, descriptive statistics were utilized for the summarization of clinical and demographic information, while continuous data were contrasted utilizing the independent t-test for data that is normally distributed or the Mann-Whitney U test for non-parametric data. Spearman's rank correlation coefficient was utilized to evaluate correlation of serum bile acids with clinical factors like Child-Pugh and MELD scores. A p-value of <0.05 was utilized as the criterion for statistical significance.

## Results:

The research included 100 participants, 50 with cirrhosis of the liver and 50 controls, to evaluate the correlation of serum bile acids with the severity of liver cirrhosis. The data were analyzed based on demographic variables, liver function tests, serum bile acid concentration, and clinical severity by the Child-Pugh and MELD scores. 12 tables have been introduced to show differences between cirrhosis and control groups as well as their correlation with liver function and severity scores. Results of these tables show that high bile acid concentrations are correlated with greater liver cirrhosis, and serum bile acid concentrations can be potential biomarkers for measuring liver function and disease severity. Table 1 (see PDF) shows the demographic profile of the participants in both groups. The age distribution was similar between the cirrhosis and control groups, with the majority of participants being in the 51-70 years age range. A slightly higher number of females were present in both groups. Table 2 (see PDF) shows and compares the liver function test results, revealing significant differences between the cirrhosis and control groups. Cirrhosis patients had elevated bilirubin levels and reduced albumin levels, consistent with liver dysfunction. Table 3 (see PDF) shows the serum bile acid levels in both groups. Serum bile acid levels were significantly higher in patients with cirrhosis, especially in those with advanced stages of the disease (Child-Pugh class C). Table 4 (see PDF) shows the correlation between serum bile acid levels and the Child-Pugh scores. A positive correlation was found between bile acid levels and the severity of cirrhosis as measured by Child-Pugh score (r = 0.76, p < 0.001). Table 5 (see PDF) shows the correlation between serum bile acid levels and MELD scores. A significant positive correlation was observed, indicating that higher bile acid levels are associated with worse liver function and higher MELD scores (r = 0.68, p < 0.001). Table 6 (see PDF) shows and correlates liver imaging findings (fibrosis and cirrhosis stage) with serum bile acid levels. Advanced fibrosis and cirrhosis stages were significantly associated with higher bile acid concentrations. Table 7 (see PDF) shows and compares serum bile acid levels across different histological stages of liver cirrhosis. Higher bile acid levels were observed in more advanced stages of cirrhosis. Table 8 (see PDF) shows the correlation of serum bile acid levels with the presence of ascites in cirrhotic patients. Patients with ascites showed significantly higher bile acid concentrations. Table 9 (see PDF) shows compares bile acid levels in patients with and without hepatic encephalopathy. Higher bile acid levels were found in patients with encephalopathy. Table 10 (see PDF) shows compares serum bile acid levels in patients on the liver transplant waiting list with those not on the list. Elevated bile acid levels were associated with a higher likelihood of being on the transplant list. Table 11 (see PDF) shows the relationship between bile acid levels and the presence of portal hypertension in cirrhotic patients. Higher bile acid levels were associated with the presence of portal hypertension. Table 12 (see PDF) shows and evaluates the association between serum bile acid levels and mortality risk in cirrhotic patients. Elevated bile acids were significantly associated with higher mortality risk.

Table 1 (see PDF) shows the demographic distribution of the study participants, highlighting age and gender differences between the cirrhosis and control groups. Table 2 (see PDF) compares liver function test results, showing significant differences in bilirubin, albumin, and prothrombin time between the two groups. Table 3 (see PDF) presents serum bile acid levels, which were significantly higher in cirrhosis patients, especially in advanced stages of the disease. Table 4 (see PDF) demonstrates a strong positive correlation between serum bile acids and Child-Pugh scores. Table 5 (see PDF) shows a similar correlation with MELD scores, indicating that higher bile acid levels are associated with poorer liver function. Table 6 (see PDF) correlates liver imaging findings with bile acid concentrations, highlighting the association of advanced fibrosis with higher bile acid levels. Table 7 (see PDF) provides data on bile acid levels across histological stages, showing higher levels in more severe cirrhosis stages. Table 8 (see PDF) links the presence of ascites with elevated bile acid levels. Table 9 (see PDF) demonstrates higher bile acids in patients with hepatic encephalopathy. Table 10 (see PDF) compares bile acid levels in patients on the liver transplant waiting list, showing that higher bile acids are associated with this group. Table 11 (see PDF) correlates bile acid levels with portal hypertension. Table 12 (see PDF) associates elevated bile acid levels with an enhanced risk of mortality. These results indicate that serum bile acids are a strong candidate for estimating the severity of liver cirrhosis and predicting clinical outcomes.

## Discussion:

The purpose of this research was to identify the association between serum bile acids and the severity of liver cirrhosis. The results validated that the level of serum bile acids was significantly higher in individuals with cirrhosis compared to controls. In addition, serum bile acids had a strong positive correlation with both Child-Pugh and MELD scores, which are commonly used to measure the severity of liver impairment. The findings imply that serum bile acid levels can be a significant biomarker for determining the severity of cirrhosis and progression of disease [9]. Bile acids play a crucial role in lipid digestion and absorption but, when built up in the blood, can be used as a marker of liver impairment, especially in cirrhosis. This study brings to light that the dysfunctioning bile acid handling and excretion in cirrhotics result in their high serum level [10]. The high levels of bile acids in serum among patients with cirrhosis, especially in the advanced cases, highlight the value of bile acids as markers of liver failure. Increased serum bile acids mirror the liver's failure to metabolize and eliminate these substances effectively and this may be directly proportional to the severity of the liver injury, an example of a non-invasive screening tool for assessing disease activity [11]. One of the key findings of this study was the association between serum bile acid level and clinical presentation of cirrhosis, such as ascites, hepatic encephalopathy and portal hypertension. They are signs of advanced liver disease and are poor prognostic indicators. The finding that patients with ascites and encephalopathy showed increased serum bile acid levels in keeping with the impression that bile acids are etiologically implicated in liver injury and deterioration of cirrhotic clinical picture [12]. In addition, the correlation of bile acid levels with portal pressure also suggests an interface for a pathophysiologic interaction in which bile acid deposition worsens vascular alterations of the liver and generates elevated portal pressure. This association emphasizes the wider role of bile acids outside its classical function in digestion and it is implied that it could also be the cause for the vascular and metabolic derangements in cirrhosis [13]. The association of rising levels of serum bile acids with rising mortality risk is clinically relevant. The hyperbile acids are common in cirrhosis and advanced disease and might be a marker of bad prognosis [14]. They were also at high risk of being listed on the waiting list for liver transplant, which further supports the suggestion that bile acids might predict outcomes. This indicates how serum bile acid concentration is a non-invasive indicator helpful in the estimation of the severity of liver cirrhosis and in patient stratification based on their disease recurrence as well as mortality [15].

The study suggests that serum bile acid levels could be useful as an efficient biomarker in monitoring liver cirrhosis. Because it is fairly easy and non-invasive to test bile acid, this can potentially complement existing devices like the Child-Pugh and MELD scores to assess the degree of cirrhosis. Application of bile acid measurement in routine clinical practice could enhance management of cirrhotic patients, especially early complication detection and evaluation of disease progression in the long term. Serum bile acids could also be used to measure the response to therapy, especially among patients on therapy aimed at improving liver function or managing complications such as portal hypertension and ascites. Several limitations have to be considered when interpreting the data from the present study. First, the sample size was small and larger, multi-center trials are needed in order to validate these findings and assess the generalizability of bile acid levels as a biomarker for liver cirrhosis. Second, the study was cross-sectional and thus no causal relationship between elevated bile acid levels and progression of disease can be established. Longitudinal cohorts must be used to ascertain the change in bile acid levels over a period of time and their prognostic ability for liver failure outcome or the need for liver transplantation. Last but not least, while serum bile acids were shown to be related to liver failure, to explain the underlying molecular mechanisms through which bile acids contribute to cirrhosis pathophysiology more research is needed.

## Conclusion:

Serum bile acid concentrations are highly increased in liver cirrhosis patients and these concentrations are related to the severity of liver cirrhosis as determined by Child-Pugh and MELD scores. Increased bile acids were also found to be related to complications like ascites, hepatic encephalopathy and portal hypertension, pointing towards their involvement in the evolution of liver cirrhosis. These data suggest that serum bile acids may be a useful biomarker for the evaluation of liver impairment and studies are needed to investigate their clinical use in the management of cirrhosis.
